# The combined effect of non-alcoholic fatty liver disease and metabolic syndrome on colorectal carcinoma mortality: a retrospective in Chinese females

**DOI:** 10.1186/s12957-018-1461-z

**Published:** 2018-08-10

**Authors:** Zhou-Feng Chen, Xiu-Li Dong, Qing-Ke Huang, Wang-Dong Hong, Wen-Zhi Wu, Jian-Sheng Wu, Shuang Pan

**Affiliations:** Department of Gastroenterology, The First Affiliated Hospital of Wenzhou Medical University, Wenzhou, 325000 Zhejiang People’s Republic of China

**Keywords:** Non-alcoholic fatty liver, Metabolic syndrome, Colorectal carcinoma, Prognosis

## Abstract

**Background:**

This research aimed to investigate whether metabolic syndrome (MetS) and non-alcoholic fatty liver disease (NAFLD) had both individual and synergistic effects on the prognosis for female colorectal carcinoma (CRC) patients.

**Methods:**

The relationship between CRC prognosis and NAFLD as well as MetS was evaluated in 764 female participants. Based on the NAFLD level, patients were divided into significant NAFLD (SNAFLD), “moderate” and “severe” level, and non-SNAFLD, “non” and “mild” level. All the patients were categorized into four subgroups according to the status of SNAFLD and MetS and then a comparison of CRC prognosis among those four groups was performed.

**Results:**

NAFLD, SNAFLD, and MetS were independent factors for CRC-specific mortality with the adjustment of age and other confounders. The hazard ratio (HR) of CRC-specific mortality in MetS (+) SNAFLD (+) group was significantly higher than that in other three groups. Relative excess risk of interaction (RERI) was 2.203 with 95% CI ranged from 0.197 to 4.210, attributable proportion (AP) was 0.444 with range from 0.222 to 0.667, and synergy index (SI) of 2.256 with 95% CI from 1.252 to 4.065, indicating SNAFLD and MetS had a significant synergic effect on CRC-specific mortality.

**Conclusions:**

SNAFLD and MetS are independent risk factors for CRC-specific mortality in females. Moreover, those two diseases have a synergistic effect on promoting CRC-specific mortality.

## Background

Reports within the past few decades indicate that the incidence of colorectal carcinoma (CRC) has remarkably climbed, making it a worldwide prevalent cancer, including Asia [1]. In China, CRC has been one of the top contributors of cancer-related death, which will lead to poor quality of life in survivors [2–5]. As mentioned in previous literature reviews, the prognosis for CRC can be influenced by a variety of clinicopathological features [5]. Also, previous studies indicate that appropriate postoperative strategies can evidently improve the prognosis for CRC [6, 7]. Therefore, it is of urgent need to determine the adverse outcome-associated predictors for CRC patients.

Non-alcoholic fatty liver disease (NAFLD), a common issue worldwide, is induced by fat accumulation in the liver [8]. Numerous studies have discovered that NAFLD can promote the development of CRC [9]. Nonetheless, few studies are available concerning the association between NAFLD and CRC prognosis. Currently, only four studies have mentioned inconsistent results [10–13]. On the other hand, metabolic syndrome (MetS) is defined as a synergic syndrome involving different metabolic dysfunctions, including central obesity, hypertension, hyperglycemia, and dyslipidemia, which is also considered as a risk factor of CRC occurrence [14]. However, the relationship between MetS and CRC prognosis remains a source of controversy. Some studies show that MetS will increase the risk of developing CRC and will lead to poor prognosis [9, 15], but other studies demonstrate that MetS exerts no apparent influence on CRC outcomes [16].

Both NAFLD and MetS are important risk factors of CRC, but the individual and synergistic effects of NAFLD and MetS on CRC prognosis remain unclear yet. Recently, NAFLD and MetS are considered to show reciprocal causality, and each singular one has perpetuating or exacerbating effect on the other [17–20]. Specifically, NAFLD is not a simple component of MetS. Besides, the synergetic effect of NAFLD and MetS has been proved in numerous diseases [21–23]. Also, it is shown in a large sample epidemiological investigation that NAFLD can only affect the cancer-specific mortality in females but not in males, indicating that a gender-dependent property may influence disease progression [13]. Therefore, to avoid the influence of gender-dependent features, only the female patients were focused in the present study. This study aimed to evaluate whether NAFLD and MetS had both individual and synergistic effects on the prognosis for female CRC patients.

## Methods

### Demographic data and laboratory measurements

CRC patients undergoing primary surgical resection from February 2007 to November 2014 at our hospital were collected. All subjects developed no distant metastasis at diagnosis. The patient exclusion criteria were as follows: (1) patients with a past cancer history and (2) those with familial adenomatous polyposis syndrome or hereditary nonpolyposis CRC. The demographic information and clinicopathological data, including body mass index (BMI), triglycerides (TGs), and other related blood parameters, were recorded in the hospital electronic medical system. Additionally, the sixth edition of the American Joint Committee on Cancer Staging Manual was employed to estimate the CRC stage.

### Assessments of NAFLD and MetS

The severity of NAFLD was determined through hepatic ultrasound scan (Siemens, Germany) by experienced radiologists who were blinded to the CRC prognosis outcomes. The diagnosis criteria were as follows: patients with mild increase in liver echogenicity, mild attenuation in the penetration of ultrasound signal, and slightly decreased lucidity of the borders of intrahepatic vascular walls and diaphragm, and were identified as mild NAFLD; while those with diffuse increase in liver echogenicity, greater attenuation in the penetration of ultrasound signal and decrease in the visualization of intrahepatic vascular walls, particularly the peripheral branches, were deemed as moderate NAFLD; and those with gross increase in liver echogenicity, greater reduction in the penetration of ultrasound signal, and poor or no visualization of intrahepatic vascular walls and diaphragm were considered as severe NAFLD [24, 25]. Among them, moderate and severe NAFLD were combined as significant NAFLD (SNAFLD).

MetS was defined with reference to the definition in Diabetes Society of Chinese Medical Association [26]. Specifically, patients presenting at least three of the following items were considered as MetS: (i) BMI of ≧ 25 kg/m^2^, (ii) patients receiving anti-hypertensive medicine treatment with (or) the systolic blood pressure (SBP) of ≧ 140 mmHg or diastolic blood pressure (DBP) of ≧ 90 mmHg, (iii) TG of ≧ 1.7 mmol/L and (or) HDL of < 0.9 mmol/L (male) or < 1.0 mmol/L (female), and (iv) fasting plasma glucose (FPG) of ≧ 6.1 mmol/L or 2-h postprandial glucose of ≧ 7.8 mmol/L.

### Follow-up

Patients should be reexamined every 3–6 months within the first 2 years after surgery, then every 6 months for the following 5 years, and every 1 year thereafter. During every follow-up, the NAFLD status was re-verified and results in the last follow-up were acquired. Moreover, imaging findings, cytology or biopsy were systematically applied to estimate the recurrence. Moreover, the overall survival (OS) and recurrence-free survival (RFS) were recorded from the date of surgery to the date of CRC-specific death/last follow-up and recurrence/last follow-up date, respectively.

This study was approved by the Ethics Committee of Wenzhou Medical University First Affiliated Hospital, and each subject had signed an informed consent for participation. Additionally, the Helsinki and Strengthening the Reporting of Observational Studies in Epidemiology (STROBE) statement was strictly observed during the whole procedures [27].

### Statistics

SPSS 20.0 software (SPSS, Chicago, IL, USA) was employed for statistical analysis. The data were expressed as mean ± standard deviation (SD) or percentages. OS and RFS rates were calculated and compared using the Kaplan-Meier survival curves with log-rank tests. All variables were initially estimated through univariate Cox proportional hazard regression analysis, and only statistically significant variables were incorporated into multivariate Cox analysis. A two-sided *p* value of < 0.05 was considered as statistically significant.

All subjects were categorized into four subgroups according to the SNAFLD and MetS status. Then, the hazard ratio (HR) was calculated in the multivariate Cox analysis after adjusted age, CEA, stage, tumor location, and tumor differentiation. Additionally, the relative excess risk due to interaction (RERI), attributable proportion (AP), and synergy index (SI) [28] were utilized to estimate the synergistic interactions of SNAFLD and MetS on CRC-specific mortality. The ranges of RERI and AP including 0 or that of SI including 1 indicated no synergistic effect, while a RERI of > 0, AP of > 0, or SI of > 1 suggested the presence of combined biological interaction.

## Results

### Prognosis related factors

Table 1 showed all the demographic and clinicopathological results. A total of 764 subjects were enrolled, including 196 (25.7%) with MetS and 568 (74.3%) with non-MetS. Specifically, there were 186 (32.7%) and 382 (67.3%) NAFLD and non-NAFLD patients without MetS, respectively. Additionally, there were 130 (66.3%) and 66 (33.7%) MetS subjects with and without NAFLD, respectively.

**Table 1:** Characteristics of participants categorized by metabolic syndrome and NAFLD status

Characteristics	Metabolic syndrome (−)		Metabolic syndrome (+)	
	NAFLD (−)	NAFLD (+)	NAFLD (−)	NAFLD (+)
Total number	382 (67.3)	186 (32.7)	66 (33.7)	130 (66.3)
Age at diagnosis (years)	50.02 ± 10.31	51.21 ± 11.96	49.92 ± 12.11	51.82 ± 12.31
BMI (kg/m^2^)	22.42 ± 4.07	22.96 ± 4.26	24.81 ± 3.49	25.92 ± 4.23
SBP (mmHg)	116.67 ± 20.27	120.58 ± 22.32	132.31 ± 20.12	133.32 ± 20.21
DBP (mmHg)	76.62 ± 10.31	74.91 ± 11.31	77.91 ± 9.91	78.31 ± 10.02
Triglycerides (mmol/L)	1.54 ± 1.87	1.62 ± 1.71	2.21 ± 1.55	2.52 ± 1.81
HDL (mmol/L)	1.20 ± 0.312	1.28 ± 0.323	1.32 ± 0.372	1.33 ± 0.298
LDL (mmol/L)	2.52 ± 1.19	2.61 ± 1.21	2.48 ± 1.02	2.50 ± 0.98
Fasting glucose (mmol/L)	5.01 ± 3.11	5.09 ± 2.76	5.39 ± 3.51	5.50 ± 3.42
CEA (ng/ml)	21.0 ± 101.4	22.7 ± 102.1	23.2 ± 98.7	22.7 ± 101.0
Differentiation				
Well/moderate	310 (81.2)	149 (80.1)	55 (83.3)	102 (78.5)
Poorly	72 (18.8)	37 (19.9)	11 (16.7)	28 (21.5)
Stage				
I	68 (17.8)	41 (22.0)	11 (16.7)	21 (16.2)
II	164 (42.9)	87 (46.7)	25 (37.8)	47 (36.2)
III	150 (39.3)	58 (31.2)	30 (45.5)	62 (47.6)
Location				
Ascending, transverse, and descending	102 (26.7)	40 (21.5)	22 (33.3)	36 (27.7)
Sigmoid	108 (28.3)	54 (29.0)	17 (25.8)	32 (24.6)
Rectum	172 (45.0)	92 (49.5)	27 (40.9)	62 (47.7)

*NAFLD* non-alcoholic fatty livers disease, *BMI* body mass index, *SBP* systolic blood pressure, *DBP* diastolic blood pressure, *HDL* high-density lipoprotein, *LDL* low-density lipoprotein

Data are expressed as mean ± standard deviation and *n* (%)

The mean follow-up duration was 21.3 ± 17.1 months. Meanwhile, 152 (19.9%) and 139 (18.2%) patients had presented CRC-induced death and recurrence, respectively. The levels of high-density lipoprotein (HDL) cholesterol and low-density lipoprotein (LDL) cholesterol, as well as tumor differentiation and stage were related to mortality in univariate Cox analysis, as shown in Table 2. Additionally, MetS, NAFLD, and moderate/severe NAFLD were also the predictors of mortality in univariate Cox analysis. Subsequently, all significant variables were incorporated into multivariate Cox analysis, the results of which indicated that MetS, NAFLD, and moderate/severe NAFLD were the independent factors associated with mortality. For recurrence, only MetS was the significant predictor in both univariate and multivariate Cox analyses (Table 3).

**Table 2:** Cancer-specific mortality

Characteristics	Univariable			Multivariable		
	HR	95% CI	*p* value	HR	95%CI	*p* value
Age (years)	1.021	0.983–1.111	0.401			
BMI (kg/m^2^)	0.932	0.821–1.120	0.212			
SBP (mmHg)	1.122	0.913–1.342	0.514			
DBP (mmHg)	0.932	0.821–1.104	0. 314			
Triglycerides (mmol/L)	0.915	0.832–1.214	0.423			
HDL (mmol/L)	1.224	1.192–1.913	0.021*	1.201	0.925–1.651	0.312
LDL (mmol/L)	1.231	1.017–1.453	0.015*	1.218	0.941–2.092	0.513
Fasting glucose (mmol/L)	1.011	0.932–1.329	0.614			
CEA (ng/ml)	1.102	0.962–1.322	0.070			
Differentiation	0.912	0.813–0.968	0.010*	0.858	0.771–0.972	0.025^*^
Location	1.225	0.832–1.564	0.243			
Ascending, transverse, and descending	1.000					
Sigmoid	1.132	0.923–1.231	0.632			
Rectum	1.342	0.831–1.532	0.452			
Stage	1.332	1.024–2.012	0.002*	1.523	1.132–2.432	0.009*
I	1.000			1.000		
II	1.028	0.821–1.223	0.298	1.131	0.882–1.251	0.328
III	2.852	1.852–3.832	0.001*	2.432	1.632–3.212	0.001*
MetS	1.621	1.221–2.133	0.007*	1.558	1.153–2.012	0.012*
NAFLD	1.544	1.031–1.893	0.010*	1.494	1.126–1.961	0.015*
Non	1.000			1.000		
Mild	1.101	0.821–1.231	0.401	1.121	0.901–1.314	0.212
Moderate	1.452	1.113–2.371	0.001*	1.501	1.161–2.481	0.010*
Severe	1.612	1.224–2.471	0.010*	1.631	1.231–2.531	0.005*

*NAFLD,* non-alcoholic fatty livers disease, *MetS* metabolic syndrome, *BMI* body mass index, *SBP* systolic blood pressure, *DBP* diastolic blood pressure, *HDL* high-density lipoprotein, *LDL* low-density lipoprotein

^*^represent the *p* value ≤ 0.05

**Table 3:** Cox analysis of risk factors associated with recurrence

Characteristics	Univariable			Multivariable		
	HR	95% CI	*p* value	HR	95%CI	*p* value
Age (years)	1.105	0.965–1.241	0.314			
BMI (kg/m^2^)	1.165	0.823–1.284	0.287			
SBP (mmHg)	1.112	0.963–1.323	0.432			
DBP (mmHg)	1.223	0.932–1.444	0.642			
Triglycerides (mmol/L)	1.013	0.862–1.152	0.426			
HDL (mmol/L)	1.104	1.013–1.324	0.023*	1.312	0.913–1.632	0.452
LDL (mmol/L)	1.342	0.982–1.552	0.313			
Fasting glucose (mmol/L)	1.122	0.951–1.302	0.524			
CEA (ng/ml)	1.123	1.032–1.452	0.014*	1.127	0.972–1.516	0.292
Differentiation	0.882	0.823–0.965	0.009*	0.904	0.832–1.176	0.128
Location	1.302	0.921–1.542	0.392			
Ascending, transverse, and descending	1.000					
Sigmoid	1.242	0.927–1.423	0.542			
Rectum	1.132	0.912–1.402	0.356			
Stage	1.322	1.095–1.932	0.011*	1.522	1.312–1.923	0.008*
I	1.000			1.000		
II	1.092	0.902–1.123	0.223	1.223	0.922–1.433	0.321
III	2.123	1.232–2.873	0.001*	1.923	1.321–2.923	0.001*
MetS	1.722	1.224–2.722	0.020*	1.821	1.164–3.121	0.010*
NAFLD	1.542	0.935–2.226	0.109			
Non	1.000					
Mild	1.097	0.913–1.198	0.312			
Moderate	1.431	0.921–2.216	0.197			
Severe	1.582	0.945–2.368	0.210			

*NAFLD,* non-alcoholic fatty livers disease, *MetS* metabolic syndrome, *BMI* body mass index, *SBP* systolic blood pressure, *DBP* diastolic blood pressure, *HDL* high-density lipoprotein, *LDL* low-density lipoprotein

*represent the *p* value ≤ 0.05

### Synergic effect of SNAFLD and MetS on CRC-specific mortality

**Fig. 1:**
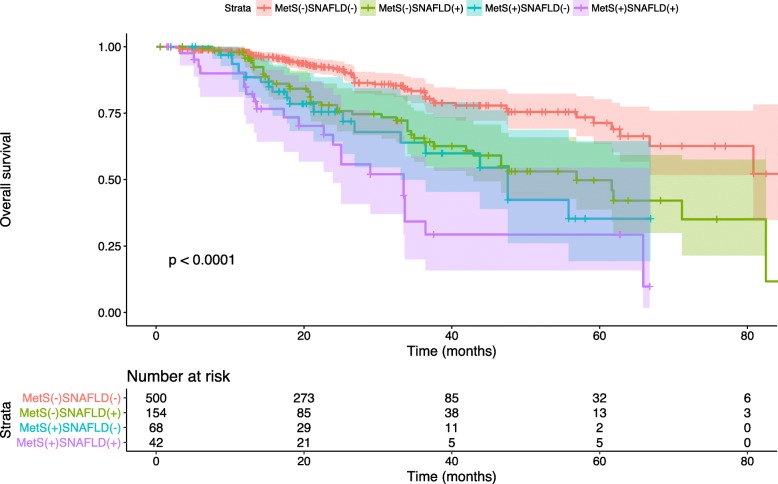
Kaplan-Meier plot indicates the overall survival in patients stratified by MetS and SNAFLD over the follow-period (*p* for log-rank test < 0.001). The light color shade surrounding each curve shows 95% CI

The above-mentioned results indicated that “moderate” and “severe” NAFLD had exerted significant positive hazard ratios (HRs) on the CRC-specific mortality, which could not be observed in “mild” NAFLD. Thus, the “non” and “mild” NAFLD were merged into the non-significant NAFLD (non-SNAFLD) group, whereas the other two subgroups, namely, moderate and severe NAFLD, were defined as SNAFLD. Thus, all patients were categorized into four subgroups according to their MetS and SNAFLD status (Table 4). After adjusting the age, CEA, stage, tumor location, and differentiation, the HRs were 1.845 (95%CI: 1.024–3.323), 1.910 (95%CI: 1.254–2.908), and 4.958 (95%CI: 2.710–9.071) for MetS(+)SNAFLD(−), MetS(−)SNAFLD(+), and MetS(+)SNAFLD(+) compared with MetS(−)SNAFLD(−), respectively. In addition, the OS rates were the lowest in MetS(+)SNAFLD(+) group compared with those in the other three groups during the follow-up period (Fig. 1). On the other hand, RERI was 2.203 (95% CI, 0.197–4.210), suggesting that the synergistic interaction had increased the relative excess risks by 2.203 times. In addition, the AP was 0.444 (95% CI, 0.222–0.667), indicating that 44.4% CRC-specific mortality was resulted from both factors contributing to the combined interaction. Additionally, the SI was 2.256 (95% CI, 1.252–4.065), suggesting that the risk of mortality in both positive patients was 2.256 times as high as the sum of risks in patients presenting only one singular factor.

**Table 4:** Interaction analysis between metabolic syndrome and SNAFLD status on mortality

Subgroup		Case	Total number	HR (95% CI)	*p* value
MetS(−)SNAFLD(−)		62 (12.4)	500	1.000	
MetS(+)SNAFLD(−)		49 (31.8)	154	1.845 (1.024–3.323)	0.012*
MetS(−)SNAFLD(+)		21 (30.9)	68	1.910 (1.254–2.908)	0.010*
MetS(+)SNAFLD(+)		23 (54.8)	42	4.958 (2.710–9.071)	0.004*
RERI	2.203 (0.197–4.210)				
AP	0.444 (0.222–0.667)				
SI	2.256 (1.252–4.065)				

*SNAFLD*, significant non-alcoholic fatty livers disease, *MetS* metabolic syndrome

*represent the *p* value < 0.05. *MetS* metabolic syndrome, *SNAFLD* significant non-alcoholic fatty liver disease

## Discussion

The underlying interaction between MetS and CRC prognosis has not been fully understandable yet. As mentioned in literature review, MetS is a well-known promoter of CRC [29–32] but is still controversial on prognosis. In our study, MetS is found to be independently associated with the recurrence and CRC-specific mortality in females, which is consistent with some previous researches. MetS is found in studies to be related to CRC mortality in both males and females [33–37]. Also, a strong relationship between MetS and recurrence has been reported in another study in both genders [15]. Moreover, Shen Z et al. [38] indicated that MetS contributed to high mortality and high recurrence in both female and male patients. However, a few results seem to be opposite, which discover that there is no association between Mets and CRC prognosis [16]. Typically, our study utilized the Chinese criterion to identify MetS, which is slightly different from the definition in other studies. In addition, most patients in the present study come from southeast China, where the diet and heredity are unique, for example, the high seafood consumption compared to other regions. It is discovered upon literature review that different MetS criteria and races can affect the outcomes [39–41], which may account for the possible explanation for those opposite results.

With regard to NAFLD, only four existing studies have concentrated on the association between NAFLD and CRC prognosis. For instance, Min YW et al. [10] indicated that there was no apparent influence of NAFLD on CRC prognosis by investigating 227 male and female participants. Oppositely, You et al. [11] enrolled 1314 male and females CRC patients and found that NAFLD was negatively related to OS. However, another study had collected 953 CRC patients and divided them into two subgroups based on the hepatic fibrosis level rather than NALFD alone. Their results demonstrated that a high hepatic fibrosis level was related to liver metastasis [12]. Additionally, Hwang YC et al. [13] investigated 318,224 Korean subjects and indicated that NAFLD was positively associated with mortality in females but not in males. Our findings indicate that NAFLD is remarkably related to mortality and not recurrence in female CRC patients. Typically, there are several differences between previous and our studies. First of all, most previous studies do not evaluate the gender-dependent issue. Except that, three out of four studies simply classify patients into NAFLD and non-NAFLD, and only one has further categorized NALFD based on the hepatic fibrosis level. By contrast, our study has divided patients into four different levels, including “non,” “mild,” “moderate,” and “severe” NALFD, and the results indicate no difference between “non” and “mild” NAFLD regarding mortality, which may partially explain the opposite results among different studies. Taken together, our study design is different from the previous ones, which may induce the different results.

The primary results demonstrate no difference between “non” and “mild” NAFLD regarding mortality. Thus, the “moderate” and “severe” NAFLD groups are merged into the SNAFLD group, while the non-SNAFLD consists of “non” and “mild” SNAFLD correspondingly. Accompanied with MetS and non-MetS status, subjects are categorized into four subgroups, and the results demonstrate that there is significant combined effect of those two factors on the CRC-specific mortality. In summary, the results have suggested a powerful synergistic interaction of those two diseases on mortality, which lead to a higher risk than that of either SNAFLD or MetS alone.

Currently, it is difficult to explain this phenomenon, but it may be partly explained by the following hypothesis. As mentioned in the literature review, MetS can induce CRC tumorigenesis and progression through multiple mechanisms, for instance, the inflammatory cytokines [42]. Nonetheless, the pathophysiological link between NAFLD and CRC prognosis remains incompletely understood. NAFLD encompasses a histological process that starts from a simple steatosis (with only fat accumulation in hepatocytes but without inflammation) to moderate and severe forms of NAFLD, such as steatohepatitis, a condition in which hepatic steatosis is accompanied by a necroinflammatory component. Inflammation is attributable to different tumor stages, which may even influence the mortality [43, 44]. Thus, the moderate or severe but not mild NAFLD may be related to the CRC-specific mortality. Taken together, there is an assumption that inflammation, induced by both MetS and SNAFLD, may be the common underlying mechanism affecting the mortality. However, this hypothesis needs to be further studied.

For the first time, this research has evaluated the synergetic influence of SNAFLD and MetS on female CRC-specific mortality. However, there are several limitations in the current study. Firstly, this is an observational study, and no results are available concerning the underlying mechanism. Secondly, the short follow-up period and data collection from only one center may weaken the clinical significance, which should be improved by extending the follow-up duration and implementing in multiple centers. Besides, to avoid gender influence, only females are investigated in the current study. Finally, a Chinese version of MetS definition, which is not worldwide applied, is applied in this study, due to the specific characteristics of patients. But this criterion has been specialized for the Chinese population and has been utilized among various diseases.

## Conclusions

It is identified in this study that SNAFLD can affect mortality but not the recurrence in female CRC patients, while MetS can affect both the recurrence and mortality. These results have complemented the field regarding the associations among SNAFLD, MetS, and prognosis in female CRC patients. In addition, the research also suggests a synergistic interaction of those two factors on the CRC-specific mortality. In general, it seems that it is urgently needed to carry out extra postoperative management to control the mortality among Chinese female CRC patients who have both SNAFLD and MetS.

## Abbreviations

(HDL) cholesterol: High-density lipoprotein
(LDL) cholesterol: Low-density lipoprotein
AP: Attributable proportion
BMI: Body mass index
CRC: Colorectal carcinoma
DBP: Diastolic blood pressure
MetS: Metabolic syndrome
NAFLD: Non-alcoholic fatty liver disease
OS: Overall survival
RERI: Relative excess risk of interaction
RFS: Recurrence-free survival
SBP: Systolic blood pressure
SI: Synergy index
TGs: Triglycerides
